# Screening Tests for Assessing Athletes at Risk of ACL Injury or Reinjury—A Scoping Review

**DOI:** 10.3390/ijerph19052864

**Published:** 2022-03-01

**Authors:** Noah Schweizer, Gerda Strutzenberger, Martino V. Franchi, Mazda Farshad, Johannes Scherr, Jörg Spörri

**Affiliations:** 1 Sports Medical Research Group, Department of Orthopaedics, Balgrist University Hospital, University of Zurich, 8008 Zurich, Switzerland; noah.schweizer@me.com (N.S.); gerda.strutzenberger@balgrist.ch (G.S.); johannes.scherr@balgrist.ch (J.S.); 2 University Centre for Prevention and Sports Medicine, Department of Orthopaedics, Balgrist University Hospital, University of Zurich, 8008 Zurich, Switzerland; 3 Motion Analysis Zurich, Department of Orthopaedics, Balgrist University Hospital, Children’s Hospital, University of Zurich, 8008 Zurich, Switzerland; 4 Department of Biomedical Sciences, Institute of Physiology, University of Padova, 35131 Padova, Italy; martino.franchi@unipd.it; 5 University Spine Centre, Department of Orthopaedics, Balgrist University Hospital, University of Zurich, 8008 Zurich, Switzerland; mazda.farshad@balgrist.ch

**Keywords:** injury prevention, knee injuries, ACL injury, physical fitness, biomechanics, risk factors, imaging, youth sport, collegiate sport, professional sport

## Abstract

Various tests are available to assess athletes for factors associated with their susceptibility and risk of anterior cruciate ligament (ACL) injury or reinjury; however, it is unclear which tests are clinically meaningful and what should be considered when using them. Therefore, the aim of this scoping review was to screen and summarize testing and to derive evidence-based recommendations for clinicians, practitioners and future research. Five databases were searched to identify studies addressing musculoskeletal morphology or functional-performance-related screening tests with a clear conceptual link or an evidence-based relationship to ACL (re)injury. A quality rating was carried out using the National Institutes of Health (NIH) Study-Quality Assessment Tool. Six different categories of common screening tests were identified: balance and postural control, gait- and running-related tests, joint laxity, joint morphology and anthropometrics, jump tests and strength tests. Predicting future injury in a complex, dynamic system based on a single screening test is methodologically challenging, which is also reflected in the highly controversial findings in the literature regarding potential associations between specific screening tests and the occurrence of ACL injuries and reinjuries. Nonetheless, various screening tests can provide clinically relevant information on ACL-(re)injury-related factors and help to provide tailored preventive measures. A selection of corresponding evidence-based recommendations is derived and presented in this scoping review.

## 1. Introduction

The social and economic burden of anterior cruciate ligament (ACL) injuries is substantial. Lifetime costs are reported at nearly $40,000 for reconstructive surgery and almost $90,000 for conservative treatment [1,2,3]. The incidence rate for ACL injuries in the general population has been reported to be 68.6 per 100,000 person-years, with particular susceptibility in male athletes in their early twenties and females in late adolescence [4]; the latter are three times more likely to suffer an ACL injury than males [5]. Hormonal, anatomical, neuromuscular and proprioceptive aspects may contribute to this sex difference [6]. Additionally, there is an increased risk of developing joint degeneration, such as osteoarthritis, later in life after ACL injury [7]. For professional athletes, an ACL injury is especially devastating, as it is often career-ending [8]. Therefore, it is crucial to regularly assess athletes at risk of ACL injury or reinjury and, most importantly, to offer them tailored preventive countermeasures [9].

Several functional-performance factors have been proposed to be associated with an increased risk of ACL (re)injury. In particular, the knee abduction angle and moment (i.e., inward movements of the knee in the frontal plane) determined by three-dimensional (3D) motion capture has been one of the main focuses of research related to ACL (re)injuries in the past [10,11,12,13]. Greater relative knee abduction angles during various dynamic movement tasks were revealed to be associated with a higher risk of injury [10], although the ability of screening tests to predict ACL (re)injuries has been the subject of substantial controversy. Other factors reported in the literature with potential associations with the occurrence of ACL (re)injuries include lower extremity (LE) or core muscle strength deficits, lack of muscle preactivity during side-cutting, LE asymmetries in jump distance or speed, lack of balance and joint laxity [14,15,16,17,18,19]. The phase of the menstrual cycle and the extended duration of the menstrual cycle also appear to influence the occurrence of injuries [20]. Of the proposed morphologic measures, some of the most frequently mentioned were tibial slope angle, femoral notch width and body mass index (BMI) [14,15,21].

However, simply demonstrating that there is an association with ACL (re)injuries does not necessarily justify the use of the test for screening with the purpose of injury prediction. To reach such a stage, several methodological challenges and validation steps must first be addressed [9]. Moreover, it is often unclear to clinicians and practitioners which tests are clinically meaningful (i.e., have a clear conceptual link or an evidence-based relationship to ACL injury or reinjury after return-to-sport) and what should be considered when using them. Comprehensive reviews on the screening of top athletes with particular emphasis on both screening tests regarding musculoskeletal morphology and functional-performance-related screening tests are, to the best of our knowledge, widely lacking.

Therefore, the aim of this scoping review was to screen the existing literature and to derive evidence-based recommendations for musculoskeletal morphology and functional-performance-related screening tests for ACL injury or reinjury in athletes that are dedicated to both clinicians and practitioners, as well as future research. As the relevant factors for primary and secondary ACL injuries, and thus also the screening approaches, are largely similar for these two contexts of application [22], we decided to address them collectively.

## 2. Materials and Methods

### 2.1. Study Design

This scoping review was reported in accordance with the Preferred Reporting Items for Systematic Reviews and Meta-Analyses extension for Scoping Reviews (PRISMA-ScR) guidelines [23]. No meta-analysis was performed for the included studies, given the heterogeneity of the different screening tests.

### 2.2. Eligibility Criteria

Studies were included if they were: (1) original, peer-reviewed and written in English; (2) enclosing athletes; (3) dealing with screening tests in the context of ACL injury prevention, return-to-sport or any other assessments of an athlete’s musculoskeletal morphology or functional performance; (4) covering screening tests with an evidence-based relationship to ACL (re)injury, or at least a clear conceptual link to ACL (re)injury; and (5) of any length of follow-up.

Studies were excluded if they were: (1) literature reviews, cross-sectional studies, conference proceedings or articles older than 25 years; (2) based on sportive subjects with nonregular training (i.e., less than three training sessions/week or recreational athletes); (3) only describing self-reported data (i.e., age, sex, sport, psychological tests or time to return-to-sport) or physiological attributes (i.e., hormones, menstrual cycle or biomarkers); (4) examining the effect of an injury prevention program or other interventions, except for interventions with an immediate effect such as perturbation; or (5) purely investigating the outcome of ACL reconstruction techniques, except studies assessing return-to-sport criteria or functional tests with a clear conceptual link to reinjury.

### 2.3. Information Sources and Search Strategy

An online literature search was carried out on 20 July 2020. The following five databases were accessed: Allied and Complementary Medicine Database (AMED) (1995-present), Embase (1995-present), Medline (1995-present), Scopus (1995-present), and Web of Science (1995-present). The following key words were included in the AMED search: test*; measur*; screen*; assess*; evaluat*; determin*; ACL; anterior cruciate ligament*; traumatic knee injur*; physical performance*; neuromuscular; control; stabilit*; valgus; abduction; rotation; hamstring*; quadriceps; core; hip*; strength*; activation*; morpholog*; architectur*; (athletic* OR sport*) injur*; risk*; predict*; prevent*; protect*; return to sport*; rehabilitation; incidence*; athlete*; player*; elite; competitive and racer*. The asterisks were used to truncate certain search terms to include similar words with different endings.

### 2.4. Study Selection, Data Collection, Data Extraction Process and Qualitative Synthesis

The eligibility assessment was performed separately by two raters in an independent standardized manner through the EPPI Reviewer Web application [24]. Duplicate articles were removed. The titles and abstracts of the retrieved studies were screened by both reviewers. Both raters then screened the full texts of the included articles for final study inclusion. Any differences in the study selection were resolved through discussion by the two raters.

A customized data extraction sheet was created and tested on 14 selected studies. The data for all eligible studies were extracted and qualitatively synthesized by Author 1. The following data items were extracted from all included studies: references, study design, sample size, sex, age, type of test, sports and major findings. The major findings of all eligible studies were extracted and categorized as follows: balance and postural control, gait and running tests, joint laxity, morphology and anthropometrics, jump tests and strength tests. These categories were then individually split into tests reporting or not reporting an association with ACL (re)injury. Based on the relative number of studies documenting an association with ACL injury, a recommendation was formulated regarding which tests to use in future injury screening. In addition, the extracted and qualitatively synthesized study content was also used to derive methodological suggestions to consider when using the corresponding screening tests.

### 2.5. Critical Appraisal of Individual Sources of Evidence (Study Quality Rating)

The quality of all selected studies was assessed using the NIH Study Quality Assessment Tools, and each paper was independently rated by Author 1 and Author 2 as *poor*, *fair* or *good* [25]. Differences in interpretation were resolved by discussion between the two raters, and consensus was achieved in all cases. Papers with *fair* and *good* ratings were included in the qualitative synthesis. For papers that were rated *poor*, consensus on final inclusion or exclusion was reached by Authors 1 and 2 based on the content.

## 3. Results

### 3.1. Study Selection

**Figure 1:**
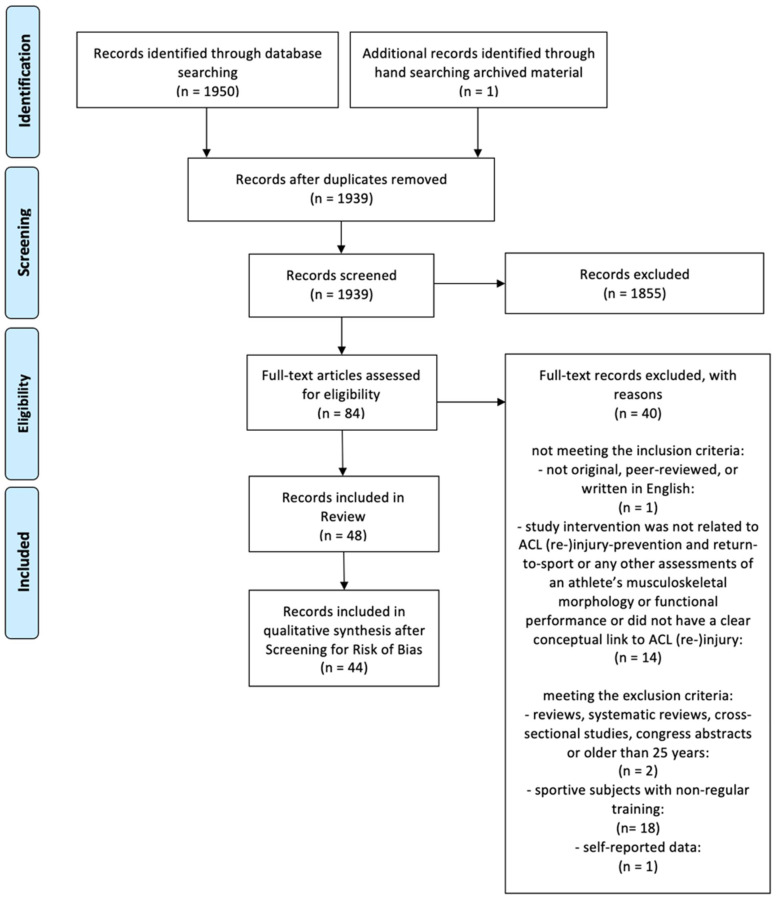
Flow chart of the study selection process.

The detailed study selection process is illustrated in Figure 1. From a pool of 1951 potential studies identified, 1939 studies were included in the screening process after duplicates were removed. Screening for title and abstract removed 1855 studies. The full-text versions of the remaining 84 studies were screened and yielded 48 studies that were included in this scoping review. The final qualitative synthesis included 44 studies after screening for risk of bias.

### 3.2. Study Quality Assessment

All studies included were rated to be of *fair* quality, with the exception of four studies (see Table 1) [26,27,28,29].

### 3.3. Results of Individual Studies

An overview of the studies included in this systematic review is given in Table 1.

#### 3.3.1. Balance and Postural Control

The first category of screening tests identified eight papers describing balance or postural control tests. A significant association between balance or postural control metrics and ACL (re)injury occurrence was documented in six papers [19,42,50,56,61,62], whereas in five papers no association was found [15,19,42,59,62]. Metrics reported to be associated with ACL (re)injury were: (1) centre of pressure (CoP) path length per time during double leg stance (injured 1.31 (0.37) cm/s; uninjured 1.15 (0.28) cm/s) [19]; (2) distance between centre of mass (CoM) and the base of support during landing after a jump task (the average CoM was 38 cm more posterior in the ACL injured athletes) [56]; (3) hip–ankle coordination during single-leg stance (M was calculated as a measure of the standard deviation of the ankle and hip angular position in the sagittal plane; M 166.2 (18.9) in controls and M 108.4 (10.1) in cases) [50]; (4) pelvic hike measured during a knee lift test (HR for high vs. low group 9.10; 95% CI 1.10–75.2) [42]; (5) lateral trunk displacement after trunk perturbation in single leg stance (OR 2.32) [61]; and (6) trunk position sense after automated structured trunk rotation in females (for each degree increase in active proprioceptive repositioning error, a 2.9-fold increase in the odds ratio of knee injury was observed) [62].

The following metrics did not show any association with ACL (re)injury: (1) CoP path length and area, which encloses the CoP movement during the double-leg stance [15,19]; (2) CoP velocity during the single-leg stance with and without movement of the contralateral limb [59]; (3) trunk position sense after automated structured trunk rotation [62]; (4) reach distance in the Star Excursion Test [59]; and (5) anterior pelvic tilt, posterior pelvic tilt, pelvic hike and pelvic drop during a knee lift test [42].

#### 3.3.2. Gait- and Running-Related Tests

Three studies assessed gait [31], agility running [39] or cutting [17]. In one case, athletes who suffered from an ACL reinjury walked with larger and more symmetrical peak knee flexion angles (pKFA) than athletes without ACL reinjury (pKFA in degrees, injured: −17.4 (8.0); reinjured: 23.3 (5.1)), indicative of a more normal gait pattern [31]. Different electromyographic (EMG) preactivity for a side-cutting task was reported in ACL-injured athletes: lower preactivity of the m. semitendinosus (21% (6%) vs. 40% (17%)) and higher preactivity of the m. vastus lateralis (69% (12%) vs. 35% (15%)) in the subsequently injured compared with the noninjured players [17]. A timed running test that evaluates agility did not show any association with ACL reinjury [39].

#### 3.3.3. Joint Laxity

Of the eight studies reporting joint laxity measures, five studies found an association with ACL (re)injury in at least one of the metrics assessed [14,15,33,46,60], while two found none [18,52]. One study was excluded from the analysis because of a potential risk of bias [27]. An association between the following metrics and ACL (re)injury was found for: (1) anterior knee stiffness (a 1 SD decrease in anterior stiffness of the knee was associated with a 2.37-fold increase in the risk of CACL injury) [33]; (2) generalized joint laxity (little finger extension and thumb opposition test were positive in the ACL injured) [14]; (3) side-to-side differences in knee laxity (for every 1.3-mm increase, the odds of ACL injury increased four-fold (95% CI, 1.68–9.69), and side-to-side difference in anterior knee laxity of 3 mm or greater had an OR of 2.4 of sustaining a contralateral ACL injury) [46,60]; and (4) decreased general joint laxity (GJL) (based on the Beigtohn GJL scale, where a larger number indicated a higher GJL; cases: 1.8 (1.3); controls: 2.7 (2.2)) [15]. No association was found for generalized laxity measures [18,46] or knee laxity in the sagittal plane [14,15,18,33,52].

#### 3.3.4. Joint Morphology and Anthropometrics

Measures of joint morphology and anthropometrics were addressed in 18 studies [14,15,18,21,26,28,29,32,33,35,37,38,39,43,44,45,51,55], of which we excluded three from the analysis because of the potential risk of bias [26,28,29]. The following metrics were the most common: four studies assessed femoral notch width [14,43,44,45], five investigated the tibial slope [21,43,44,51,55], and another five examined height and weight [15,33,35,38,39]. The following metrics were associated with ACL (reinjury): (1) decreased femoral notch width in females (12 mm and 13 mm in the twins compared to a reported mean notch of 15 (2.7) mm in a similar athletic population; 1 SD in femoral intercondylar notch width was associated with increases in the risk of suffering a CACL injury (HR = 1.88 and 2.05, respectively); every 1 mm increase in notch width at the anterior attachment of the ACL was associated with a 28% decrease in the risk of ACL graft rupture) [14,43,44] and (2) increased tibial slope measures (measured by using the angle between the longitudinal tibial axis and the line fitting the lateral tibial plateau; cases, 9.5° (3.0°) and controls, 5.6° (1.9°); every degree increase in the lateral tibial plateau slope was associated with a 32% increase in risk of ACL injury) [21,51].

One study showed that in males, the following measures were associated with ACL reinjury [43]: (1) increased posterior–inferior-directed slope of the articular cartilage in the lateral tibial plateau measured at two locations (1° was associated with a 39% increase in risk of ACL reinjury); (2) increased volume in the medial tibial spine (every 100 mm^3^ increase was associated with a 45% increased risk of ACL reinjury); and (3) anteroposterior length of the medial tibial spine (every 1 mm increase was associated with a 34% increase in the risk of ACL reinjury). Moreover, an increased distance of the lateral tibial spine was associated with ACL reinjury (every 1 mm increase was associated with a 119% increase in the risk of ACL reinjury). In females, the following metrics were associated with an increased risk of reinjury [43]: (1) decreased volume and height of the medial tibial spine (an increase in medial tibial spine volume of 100 mm^3^ was associated with a 55% decrease in risk of ACL reinjury; a 1 mm increase in the superior–inferior height of the medial tibial spine was associated with a 54% decrease in risk of ACL graft injury); (2) decreased slope of the lateral tibial subchondral bone (every 1° increase was associated with a 28% decrease in risk of suffering an ACL reinjury); (3) decreased height of the posterior horn of the medial meniscus (every 1 mm increase was associated with a 91% decrease in risk of ACL reinjury); and (4) decreased intercondylar notch width (every 1 mm increase in notch width at the anterior attachment of the ACL was associated with a 28% decrease in the risk of ACL reinjury).

Last, the posterior tibial slope, which was measured using conventional radiographs using the angle between the tibial mid-diaphysis line and the line between the anterior and posterior edges of the medial tibial plateau, was measured as the tibial slope and was associated with ACL injury (OR 5.62 for ACL injury risk if the tibial slope was over the group mean) [55]. Metrics without significant associations were height and weight [15,33,35,38,39], decreased femoral notch width in males [43,45] and lateral tibial slope [44].

#### 3.3.5. Jump Tests

The following tests were commonly investigated for a potential association with ACL (re)injury: dynamic knee valgus during drop vertical jump (DVJ) [10,11,12,13,14,35,40,48,57], maximal height at DVJ [14,16] and single-leg hop test for distance [18,30,39]. Other tests commonly used were the distance of a triple crossover hop [60], standing long jump [30,54] and jumping single-legged backwards, forwards, medially and laterally onto a force platform [34]. Furthermore, a jump coordination test for speed, counter movement jump for height and specific counter movement jump for height with restricted ankles were reported [16]. The distance of a single-leg, five-hop jump and the number of repetitions within 90 s of high-box jumping were further tests identified [54]. Last, a hop test was reported in which the subject had 30 s to jump clockwise into and out of a square as many times as possible, as well as timed sideways hopping 10 times [18].

In 10 studies, at least one association between jump tests and ACL (re)injury was stated [10,11,12,14,16,18,34,41,48,49]. Another eight did not show any association with ACL (re)injury [13,30,35,39,40,54,57,60]. The main metrics associated with ACL (re)injury were knee abduction moment (KAM) or knee abduction angle (KAA) during DVJ (KAA at landing was 8° greater in ACL-injured than in uninjured athletes; KAA was increased at one knee in twin sisters—sibling 1, −11.3°; sibling 2, −7.7°—versus controls, 24.6° (5.6); dynamic knee valgus was significantly greater in the injured group than in the control group at ground contact (2.1 (2.4) vs. 0.4 (2.2) cm) [10,14,48]. Further associated metrics were stiff DVJ landing with less hip flexion (HR for each 10° increase in hip flexion, 0.61 (95% CI, 0.38–0.99)), greater peak external knee flexion moment (HR for each 10-Nm increase in knee moment, 1.21 (95% CI, 1.04–1.40)) and a higher Landing Error Scoring System (LESS) score at DVJ (uninjured athletes had lower LESS scores (4.43 (1.71)) than injured athletes (6.24 (1.75)) [41,49]. The reactive strength index (RSI) in men (calculated by dividing the jump height (mm) of the first jump by the ground contact time (ms)) [16], the side-to-side differences in a single-leg maximal hop test (36% of injured athletes had an unequal side-to-side performance > 10 cm, whereas only 23% of uninjured athletes had an unequal performance) [18] and the time to stabilization measured by jumping onto a force platform (injured compared with uninjured athletes took 1.58 (0.39) and 1.09 (0.52) seconds to stabilize, respectively) [34] were also reported to be associated with ACL injury.

In partial contradiction to these studies, other studies revealed no associations with ACL (re)injuries for the following metrics: (1) the KAM or knee abduction angle during DVJ [11,12,13,35,40]; (2) the LESS score for DVJ [57]; (3) single-leg hop and standing long jump scores normalized to athlete height [30]; (4) maximal jump height [14]; (5) side-to-side differences for distance in single-leg hop tests [39,60]; and (6) maximal triple crossover hop distance [60]. Additional metrics with no associations included RSI in females, speed at the jump coordination test, height at the counter movement jump and height at a specific counter movement jump variation with restricted ankles [16]. The distance of a single-leg, five-hop jump and standing long jump, as well as the repetitions of high-box jumping within 90 s, were also not associated with ACL (re)injury [54]. The same applies to a hop test in which the subject had 30 s to jump clockwise into and out of a square as many times as possible and to hop sideways 10 times [18].

#### 3.3.6. Strength Tests

Eleven studies assessed strength measures [14,15,16,33,36,38,39,47,53,54,58]. An association between ACL (re)injury and strength metrics was observed for: (1) increased hip abduction strength (cases, 1.4 (0.3) Nm/kg; controls, 1.2 (0.2) Nm/kg) [15]; (2) decreased hip abduction strength (hip strength as percentage of body weight; HR 1.80 (95% CI, 1.03–3.16) for 1 SD decrease in maximal hip abduction strength; cases, 30.8 (8.4); controls, 37.8 (7.6)) [36,38]; (3) decreased hamstring to quadriceps strength ratio at 60°/s, but not at 180°/s or 300°/s (HR 10.6 per 10% difference; 95% CI, 10.2–11) [39]; (4) decreased hip external rotation strength (cases, 17.2 (2.9)); controls, 22.1 (5.8) as a percentage of bodyweight) [38]; (5) one-repetition maximum (1RM) barbell squat (a relative 1RM squat smaller than or equal to 105% of bodyweight had an OR of 7.64 (95% CI, 1.60–36.52) for ACL injury) [53]; and (6) the ratio of flexion to extension in a core strength test (CST)—in males only (statistics only available for separate age groups) [16]. The same study reported the relative CST extension force, the relative CST flexion force and the reactive strength index during DVJ (all in males), as well as the absolute CST extension force and the absolute CST flexion force in females to be associated with ACL (re)injuries (statistics only available for different age groups) [16].

In partial contradiction to these studies, other studies reported no association with ACL (re)injury for: (1) absolute hamstring and quadriceps strength [15,33,36,40,48,59]; (2) hamstring-to-quadriceps strength ratio [14,15,36,39,58]; (3) the 1 RM leg press strength [36,58]; (4) hip abduction, hip adduction, hip internal rotation, hip external rotation, trunk flexion, trunk extension, ankle dorsiflexion and ankle plantar flexion strengths [33,58]; and (5) work fatigue in isokinetic hamstring and quadriceps strength [39]. Furthermore, a timed jump-coordination test in which subjects had to complete a course as fast as possible, absolute and relative leg force, as well as the ratio of absolute left to right leg force in the unilateral leg press strength test, showed no association with ACL (re)injury [16]. The same study revealed no association with absolute CST flexion and extension force in males in CST, relative CST flexion and extension force or ratio of absolute flexion to extension force in females [16]. The same applied to the core-to-leg strength ratio, jump height in a counter movement jump (with and without restricted ankles), jump height and contact time during a DVJ test, reactive strength index during a DVJ test in females, number of jumps during a strength endurance test, line run test index where medicine balls had to be touched as fast as possible while sidestepping and distance in the Cooper test [16]. An assessment for timed push-ups was carried out in one study, but again showed no association [54].

### 3.4. Study Populations

Of our literature search that included 44 articles for final analysis, 20 studies analysed a female-only population [10,11,12,13,14,15,17,19,30,31,33,35,40,41,44,46,48,50,58,59], four reported a male-only population [39,45,51,55] and 20 studies investigated a mixed population [16,18,21,32,34,36,37,38,42,43,47,49,52,53,54,56,57,60,61,62]. Regarding sport disciplines, there were 26 studies that included basketball athletes [10,11,14,15,19,21,30,33,34,35,36,37,38,40,41,43,44,45,46,47,48,52,53,56,57], 26 that included football (soccer) [10,12,13,14,17,21,30,33,34,35,37,38,39,40,43,44,46,47,49,52,53,55,56,57,58,59], 12 that included handball [12,13,17,19,38,39,48,52,53,56,58,59] and nine that included volleyball [10,12,21,30,34,35,38,40,57]. Other sports that were mentioned less frequently were American football [21,34,43,51,56,57], field hockey [33,34,35,43,44,57], lacrosse [33,34,35,43,44,57], skiing [16,18,21,52,53,54], floorball [11,36,41,42,53], rugby [35,43,52,57], ice hockey [21,52,53], track and field [33,43,44], gymnastics [35,57], wrestling [21,43], futsal [38], martial arts [21], motocross [52], softball [43] and ultimate frisbee [35].

## 4. Discussion

The goal of this scoping review was to identify meaningful musculoskeletal morphology and functional-performance-related screening tests to identify and assess at-risk athletes for factors associated with anterior cruciate ligament (ACL) injury or reinjury. The tests were grouped into six basic categories: balance and postural control, gait- and running-related tests, joint laxity, joint morphology and anthropometrics, jump tests and strength tests. Some studies applied tests of more than one category, which is why some studies are referenced in multiple sections.

At this point, the difference between the concept of injury association and injury prediction should be highlighted. The observation of a certain association between the result of a screening test and a subsequent injury does not necessarily mean that such a test can be used for the purpose of predicting injury (or identifying athletes at particular risk of injury). In a complex and multifactorial system, such as that of injury causation, there may remain some risk of bias from unknown contributing factors, which certainly limits the conclusions that can be drawn regarding cause and effect. Furthermore, because the morphological or functional metrics tested may be subject to a dynamic and constantly evolving process (e.g., athletes’ strength abilities may have changed from their assessment at baseline to the onset of injury), the predictive value of a single parameter based on linear statistics is severely limited.

There are established reporting standards for predictive modelling, such as the TRIPOD statement [63]. Moreover, it has been proposed that the following three steps would be required for injury prediction [9]: first, a direct association between a screening-test marker and injury risk must be documented, and appropriate cut-off values need to be derived. Second, acceptable diagnostic test proprieties must be demonstrated, and first associations and predefined cut-off values need to be verified in multiple cohorts. In a final, third step, it would need to be shown that a corresponding screening and intervention program is more beneficial than one given to all athletes regardless of their individual risk [9]. Such a screening test could then be used for the purpose of injury prediction.

In view of these various methodological challenges in “predicting” future injuries with complex and multifactorial causation within a constantly changing dynamic system [9], an alternative approach could be to offer preventive countermeasures to all athletes, but tailor them individually based on their screening test results. This is the context of application the current review refers to.

### 4.1. Balance and Postural Control

Postural control or balance has been described as the ability to maintain an upright stance by dynamically integrating internal and external forces as well as environmental factors [64,65]. The sensory input used for postural control originates mainly from the vestibular system of the inner ear, vision and proprioception, with the latter playing the most crucial role for balance [65]. Proprioception has been explained as encompassing the sensations of joint motion and position [64]. Failure to coordinate different body parts may put the knee in high-risk positions [50]. Accordingly, the assumption that better balance leads to fewer compensating movements and decreases the load on the knee joint in unfavourable positions during highly dynamic tasks seems reasonable. This theory is supported by successful neuromuscular training and balance interventions [66,67,68].

The majority of studies that assessed balance revealed an association with ACL (re)injury [19,42,50,56,61,62]. However, measures of balance considerably differed between the tests, with only two studies following similar testing protocols [15,19]. Tests that examine knee kinematics and kinetics during DVJ may also indicate balance deficits. These are, however, discussed separately in the section on jump tests. Commonly used balance and postural control screening tests range from single- or double-leg stance on a force platform [15,19,59], tracking the motion of single body segments [42,50] or considering the entire body [56]. Most typically, two- and three-dimensional camera systems and static or dynamic movement tasks as well as specific testing devices (e.g., for sensing the trunk position) were used [61,62]. Force platforms provide information about CoP pathways during stabilization tasks (e.g., overall balance deficits) but not about the causative specific body structures and their movement patterns [15,19,42,59]. Current research is therefore increasingly broadening its focus from the assessment of overall balance to a more specific description of whole-body kinematics during dynamic movements such as side-cutting or change-of-direction tasks [61,62,69,70].

Based on the heterogeneity of the test designs used and, therefore, the results obtained, we do not currently recommend including balance measures in standard screening tests of athletes. However, we encourage further research because of the plausible links mentioned above.

### 4.2. Gait- and Running-Related Tests

Three tests were found that deal with gait- and running-related movements in the horizontal plane, namely, gait assessment [31], the agility run test [39] and the side-cutting test [17]. One study investigated primary ACL injury [17], while the other two assessed ACL reinjury [31,39]. An association between walking with larger and more symmetrical peak-knee-flexion angles, which are indicative of a more normal gait pattern, and ACL reinjury was described by Capin et al., 2017 [31]. This may be explained by the earlier return-to-sport clearance in the reinjured group, implying that the time until return-to-sport is a more meaningful marker for reinjury than knee function [31]. However, due to the small number of studies, more research is needed to conclude whether such tests should be used for athlete screening in the context of ACL injury or reinjury after returning to sports.

From a purely methodological point of view, we strongly recommend the use of three-dimensional instead of two-dimensional assessment methods, as movements relevant to ACL (re)injuries mainly occur in the anatomical sagittal plane and not in the global frontal plane. Moreover, when assessing gait- or running-related direction changes, based on the current body of knowledge, side-cutting, rather than sidestepping, should be favoured, as there is more evidence that the first is associated with ACL (re)injuries, as described in the Section 3.

### 4.3. Joint Laxity

Joint stabilization has been described to be composed of active (dynamic knee alignment) and passive (ligaments and tendons) components [46]. One advantage of assessing passive joint constraint is its relative ease of measurement. Commonly, this can be done with the use of goniometers, without the need for a laboratory or portable arthrometers, to examine anteroposterior knee laxity [33,46]. An ACL with an increased ability to resist anterior displacement is less likely to tear [33]. Moreover, the shear forces at the joints differ depending on the cocontraction of the muscles associated with each joint [71]. The forces acting on the passive joint constraints during movement are considered in kinetic and kinematic knee tests, as described in our section on jump tests.

Only one of the seven studies we included for our analysis found an association between ACL (re)injury and sagittal plane knee laxity by measuring tibiofemoral translation with a knee arthrometer [60]. Two studies reported an association in side-to-side differences in knee laxity and ACL injury [46,60] compared to five studies reporting no association of knee laxity in the sagittal plane [14,15,18,33,52]. Therefore, if using such tests at all, we recommend using side-to-side comparisons of knee laxity over absolute measures. Since ACL injuries occur more frequently in females during puberty, sex-specific hormonal changes that increase joint laxity in females may explain this effect [46]. Only three of the studies analysed included male and female athletes [18,52,60]. Meanwhile, the other four studies examined only female populations [14,15,33,46].

### 4.4. Joint Morphology and Anthropometrics

Although it is difficult to alter the morphologic factors associated with ACL injury, their identification is nevertheless desirable to develop tailored prevention programs for factors that can be influenced in order to compensate for those that cannot. Among the 15 studies identified as part of this review, there was a large heterogeneity in the metrics assessed [14,15,18,21,32,33,35,37,38,39,43,44,45,51,55]. It seems well established that ACL (re)injury is not related to weight or height, as all five studies that assessed these metrics showed no association [15,33,35,38,39]. Four studies reported an association between reduced femoral notch width and female sex; however, only one of these studies analysed both sexes [43], while the other three only looked at one [14,44,45]. Tibial slope was measured differently in all studies, and the results were likewise different [21,43,44,51,55].

Overall, it seems that screening female athletes for femoral notch width may help identify athletes at risk. However, magnetic resonance imaging (MRI) scans are expensive, and computed tomography (CT) scans are potentially harmful because of the radiation emitted. Standardized routine MRI examination of the femoral notch width might therefore only be indicated in female athletes who are at increased risk. Moreover, although with less evidence, we also recommend testing other measures, such as tibial slope, in both sexes. In this context, future research should focus on improved (e.g., real 3D) or alternative (less cost intensive or more mobile) imaging modalities to assess geometric knee characteristics. Such modalities may help to better scale up the application of joint morphological screening in a wider mass of athletes.

### 4.5. Jump Tests

For more than 15 years, attempts have been made to predict future ACL (re)injuries based on lower-limb biomechanical data by identifying deficient movements in laboratory settings [10]. The knee abduction moment (KAM) is most frequently studied since dynamic knee valgus has been reported to increase the load on the ACL [10]. While 3D kinematic measures are considered to be the gold standard for the assessment of KAM or other dynamic knee-valgus-related estimates [72], for two-dimensional (2D) kinematic measurements, a moderate to high correlation has been reported [72,73]. We found slightly more studies that looked at three-dimensional (3D) measures [10,11,12,13,14,41] than 2D measures [35,40,48,49,57].

Overall, the assessment of KAM or other dynamic knee-valgus-related estimates during DVJ seems to be an intuitive approach, as it resembles the movement patterns of typical ACL injury mechanisms [74,75,76]. However, as reported above, the majority of studies reporting KAM during DVJ failed to show a positive association with ACL (re)injury. Thus, when aiming to predict ACL injuries (i.e., identify athletes at risk), dynamic knee-valgus-related screening tests may not be clinically helpful [77]. Nevertheless, the detection of individual functional deficits in dynamic leg-axis stability (e.g., leg asymmetries) can be recommended for periodic athlete examinations because they can provide clinically relevant information to better tailor injury prevention or rehabilitation programs to the needs of individual athletes and to track their progress during training or return to sports. A promising secondary investigation that could be conducted concurrently with DVJ testing is the evaluation of athletes’ landing strategies. In this context, it has been suggested that stiff landings with less hip flexion are associated with an increased risk of ACL injury [11,41]. In addition, timing-based assessments of muscle activation before and during jump landing may provide promising alternative screening metrics. Indeed, poor neuromuscular control may lead to inefficient movement strategies by increasing ACL loading. Accordingly, previous studies have used alternative drop jump testing protocols to analyse muscle activation of the knee stabilizers to identify a potential risk of dynamic valgus due to inadequate neuromuscular timing [78,79]. Reviewing jump tests other than those related to dynamic knee valgus, we found that all but one test fit into one of two categories: the first category assessed the asymmetry between legs in single-leg jumps, focusing on metrics such as jump height or width [18,39,60]. For this type of test, only one study found a positive association with ACL (re)injury [18]. The second category compared the bilateral jump height or distance between players with and without ACL (re)injury [14,16,18,30,54] and revealed a positive correlation in only one study [16]. Therefore, it seems that measurements such as distance or height jump are not a promising way to identify at-risk athletes. The only test that did not fall into either category examined balance by jumping on a force platform and found a positive association with ACL injury [34].

A further development of 3D measurements in the laboratory would be the analysis of injuries or high-risk situations during a game with the help of wearable sensor technology, which assesses relevant information almost in real time [80]. Although not yet fully reliable [80], it is likely that such technologies (coupled with deep-learning algorithms or combined with computer-vision approaches) will improve the understanding of movements associated with ACL (re)injury in the near future. As only two studies investigated male athletes performing a DVJ task, we recommend further research including both sexes [49,57]. Finally, timing-related assessments of muscle activation prior to and during jump landings may provide promising alternative approaches

For meaningful testing, we recommend not only the inclusion of 3D kinematics and kinetics for DVJs but also the consideration of more complex jumping tasks that require multiplane body movements, including rotational components or single-leg landing strategies. Generally, we recommend choosing DVJ instead of CMJ because no positive correlation was found between CMJ and ACL (re)injury.

### 4.6. Strength Tests

The relationship between reduced overall lower extremity or trunk strength and ACL injury has been explained by several mechanisms, one of which is impaired neuromuscular control [36]. Other reported mechanisms were impaired muscle recruitment, decreased hamstring strength as an ACL-synergist and trunk control impairments [36]. Finally, increased knee valgus was presumed to be a result of hip abductor weakness [36]. Conversely, it has been suggested that superior lower-extremity strength may lead to ACL (re)injury, as strong players spend more time on the pitch and are able to run and change direction faster [36].

Stabilization of the knee joint is primarily achieved by cocontraction of the hamstring and quadricep muscles [47]. A relative increase or time shift in generated hamstring activation and force compared to that of the quadriceps during jump landings reduces the strain on the ACL [81,82,83]. We found one study that reported an association between the hamstring and quadricep strength ratio and ACL injury [39], while five studies showed no association [14,15,36,39,58]. However, inadequate muscle activation patterns could also lead to ACL (re)injury [58]. In this context, it should be emphasized that the muscle cocontraction patterns during dynamic movements in a functional situation such as a drop jump are expected to give a more complete idea of the ACL-(re)injury-relevant thigh muscle function than the hamstring-to-quadriceps strength ratios in isolated situations alone. Accordingly, we also recommend assessing EMG activity during dynamic movements and focusing on intermuscular activation patterns rather than just standard strength tests.

The previously reported associations between low hip abduction, hip external rotation strength force and the risk for ACL injuries [36,38] can theoretically be explained by weak hip muscles not being able to eccentrically counteract the hip adduction and internal rotation of the femur as part of the dynamic knee valgus motion during landings. In contrast, another study by Shimozaki and colleagues reported greater, not less, hip abductor muscle strength to be an ACL injury risk factor [15]. They argued that during sporting activities, athletes who have greater hip abductor strength may compensate for this and therefore tend to adduct the hip, which may induce greater knee valgus motion [15]. Because of this controversy, the current state of scientific knowledge remains inconclusive. Nevertheless, corresponding screening tests may provide knowledge of specific deficits that can then be addressed with tailored interventions and may help to track any progress during training or return to sport.

### 4.7. Study Populations

The bias in the more frequent reporting of female populations could be explained by their relatively higher risk of suffering an ACL injury. We recommend that future studies include both male and female athletes to better understand sex differences in the association of screening tests with ACL (re)injuries.

### 4.8. Potential Recommendations for Clinicians and Practitioners and Future Research

Based on the current evidence summarized in this review, the following recommendations can be derived for clinicians and practitioners and future research regarding musculoskeletal morphology or functional-performance-based screening tests to assess athletes at risk of ACL injury or reinjury. Table 2 presents the different test categories with recommendations for clinical use, including application aspects and current limitations/future research proposals.

### 4.9. Methodological Considerations

One limitation is the heterogeneous reporting within and between the reviewed studies. Within similar tests, different metrics were measured, and similar metrics were often measured differently. This may explain why there was no strong evidence for any of the metrics assessed. The quality of the reviewed studies was *fair,* with none of the studies fulfilling all criteria to be rated *good*. The search was restricted to English studies, which might have created bias. Our search was limited only to elite, nonrecreational athletes with regular training, potentially neglecting valuable studies with less-trained subjects on the borderline between competitive and recreational sports. Due to inconsistent reporting on follow-up times and rates, we decided not to include these metrics. This may introduce bias by not including injuries that occurred with a longer delay to the screening test. Because of the heterogeneous reporting and small number of studies looking at the same screening tests, we did not perform subgroup analyses for sex and sport.

## 5. Conclusions

The ability to predict injuries is limited by the fact that the test results of the injured and uninjured groups cannot be clearly distinguished by means of predefined thresholds. However, despite not being able to accurately predict injuries, screening athletes for ACL-(re)injury-relevant factors tracks their progress during training or return-to-sport and, most importantly, offering them tailored preventive countermeasures is still possible by combining different screening tests. Recommendations on which screening tests should be considered for use by clinicians and practitioners in their daily routines and what should be considered when using them have been derived as part of this scoping review. (Table 2). Finally, potential targets for future research are suggested.

## Figures and Tables

**Table 1:** Overview of the studies included in the systematic review: references, study design, sample size, mean age, type of test, sport, quality rating and major findings.

References	Study Design	Sample Size	Age, Mean (SD)	Type of Test						Sport						Quality Rating	Major Findings
				Balance/Postural Control	Gait and Running Tests	Joint Laxity	Morphology and Anthropometrics	Jump Tests	Strength Tests	Basketball	Football (Soccer)	Handball	Volleyball	Other	NR		
Amraee (2013) [26]	Case–control	n = 106 males	cases: 24.98 (4.83); controls: 24.62 (4.46)				X			X	X	X	X			poor	Tibial torsion, hip internal rotation, ankle dorsiflexion, navicular drop and hip anteversion, but not Q angle, hip external rotation and knee hyperextension were risk factors for ACL injury.
Brumitt (2019) [30]	Prospective cohort	n = 360 females	19.3 (1.4)					X		X	X		X			fair	Performance in single-leg hop and standing long jump did not predict ACL reinjury
Capin (2017) [31]	Case–control	n = 14 females	16.1 (1.7)		X										X	fair	ACL-injured athletes had higher BMI and walked with larger and more symmetrical peak knee flexion angles, indicative of normal gait patterns
Carter (2017) [32]	Case–control	n = 176; 54 males, 122 females	NR				X								X	fair	Increased internal rotation position of the tibia was associated with increased risk of ACL injury, while extensor moment arm of the knee, coronal patellar tendon angle and sagittal patellar tendon angle were not
Davey (2019) [33]	Case–control	n = 109; 48 males, 61 females	17.1 (2.14)			X	X		X	X	X			X		fair	Younger age, decreased anterior stiffness of the contralateral knee and increased hip anteversion were associated with a contralateral ACL injury
DePhillipo (2019) [21]	Prospective cohort	n = 245 mixed sexes	NR				X			X	X		X	X		fair	An increase in lateral posterior tibial slope was associated with ACL injury, while there was no association for sex, age or body mass index
DuPrey (2016) [34]	Prospective cohort	n = 278 mixed sexes	18.5 (0.9)					X		X	X		X	X		fair	ACL injured athletes took longer to stabilize during backwards jump landing than uninjured athletes
Goetschius (2012) [35]	Case–control	n = 1855 females	cases: 17.8 (1.8); controls: 18.1 (1.7)				X	X		X	X		X	X		fair	PKAM was not associated with ACL injury
Gomes (2014) [27]	Prospective cohort	n = 55 males	25.8 (4.4)			X					X					poor	There was lower hip rotation in the rerupture group.
Hägglund (2016) [28]	Prospective cohort	n = 4556 females	14.1 (1.2)				X				X					poor	Significant predictor variables were age >14 years, knee complaints at the start of the season and familial disposition of ACL injury
Hewett (2005) [10]	Prospective cohort	n = 205 females	cases: 15.8 (1.0); controls: 16.1 (1.7)					X		X	X		X			fair	Knee motion and knee loading during a landing task were associated with ACL injury in female athletes
Hewett (2010) [14]	Case–control	n = 2 females	16 (0)			X	X	X	X	X	X					fair	Twins that suffered an ACL injury had potential risk factors including: increased knee abduction angles, decreased knee flexion angles, increased general joint laxity and decreased femoral intercondylar notch width
Hietamo (2020) [36]	Prospective cohort	n = 403; 214 males, 189 females	16.0 (1.6)						X	X				X		fair	Decreased hip abduction strength was associated with ACL injury
Jenkins (2007) [37]	Case–control	n = 105 mixed sexes	19.1 (1.6)				X			X	X					fair	Subtalar joint neutral position and the navicular drop test were not associated with ACL injury
Khayambashi (2016) [38]	Prospective cohort	n = 501; 363 males, 138 females	cases: 21.8 (4.2); controls: 21.3 (5.2)				X		X	X	X		X	X		fair	Hip abduction and external rotation strength were associated with noncontact ACL injury
Krosshaug (2016) [13]	Prospective cohort	n = 782 females	21 (4)					X			X	X				fair	VDJ tests in uninjured athletes were not associated with ACL injury
Kyritsis (2016) [39]	Prospective cohort	n = 158 males	cases: 22 (5); controls: 21 (4)		X		X	X	X		X	X			X	fair	Athletes who did not meet the discharge criteria before returning to sport had a four-fold greater risk of sustaining an ACL graft rupture compared with those who met all six discharge criteria
Landis (2018) [40]	Prospective cohort	n = 187 females	19.5 (1.21)					X		X	X		X			fair	The FMS™ was associated with ACL injury
Leppanen (2017) [41]	Prospective cohort	n = 174 females	15.4 (1.9)					X		X				X		fair	Landing with less hip flexion and greater peak external knee flexion moment was positively associated with ACL injury
Leppanen (2017) [11]	Prospective cohort	n = 174 females	15.4 (1.9)					X		X				X		fair	Stiff landings in a vertical-drop jump test were positively associated with ACL injury
Leppanen (2020) [42]	Prospective cohort	n = 319 mixed sexes	16.0 (1.9)	X						X				X		fair	High lateral pelvic hike angles were associated with ACL injury in a high-risk vs. low-risk group
Levins (2016) [43]	Case–control	n = 69 mixed sexes	**				X			X	X			X		fair	In females but not in males there was an association between a decrease in femoral intercondylar notch width, as well as a decrease in height of the posterior medial meniscus, and ACL graft rupture
Levins (2017) [44]	Prospective cohort	n = 62 females	NR				X			X	X			X		fair	After ACL injury, subsequent injury to the contralateral ACL was associated with decreases of femoral intercondylar notch width, mediolateral width of the lateral tibial spine, height of the medial tibial spine and thickness of the articular cartilage located at the posterior region of the medial tibial compartment
Lombardo (2005) [45]	Case–control	n = 305 males	NR				X			X						fair	Intercondylar notch width was not associated with ACL injury
Miljko (2012) [29]	Case–control	n = 51 females	cases: 21; controls: 17				X					X				poor	The inner angle of the femur condyles is higher and the intercondylar notch width is smaller in athletes with ACL tear
Myer (2008) [46]	Prospective cohort	n = 1558 females	cases: 16.3 (1.7); controls: 15.6 (1.4)			X				X	X					fair	An increase in knee hyperextension was associated with ACL injury
Myer (2009) [47]	Prospective cohort	n = 132 mixed sexes	NR						X	X	X					fair	Decreased hamstring strength but not quadricep strength was associated with ACL injury in female athletes
Numata (2018) [48]	Prospective cohort	n = 291 females	15 (0)					X		X		X				fair	Dynamic knee valgus was associated with ACL injury
Oshima (2018) [19]	Prospective cohort	n = 287 females	15 (0)	X						X		X				fair	Balance was associated with noncontact ACL injury
Padua (2015) [49]	Prospective cohort	n = 829 mixed sexes	13.9 (1.8)					X			X					fair	Noninjured participants had lowered LESS scores than injured participants
Paterno (2015) [50]	Prospective cohort	n = 61 females	cases: 15.4 (0.5); controls: 17.2 (0.6)	X											X	fair	Hip–ankle coordination was altered in female athletes who sustained a second ACL injury after return-to-sport
Rahnemai-Azar (2016) [51]	Case–control	n = 90males	20 (2)				X							X		fair	Increased tibial plateau slope is associated with ACL injury
Raschner (2012) [16]	Prospective cohort	n = 370 mixed sexes	NR					X	X					X		fair	Core strength was associated with ACL injuries
Rosenstiel (2019) [52]	Retrospective cohort	n = 72 mixed sexes	23.2 (NR)			X				X	X	X		X		fair	No association was found for knee laxity in the sagittal plane and ACL reinjury
Ryman (2017) [53]	Prospective cohort	n = 225 mixed sexes	males: 17 (0.8); females: 17 (1)						X	X	X	X		X		fair	The odds of sustaining an ACL injury increased in the weak 1RM barbell squat group compared with the strong group
Schmitt (2016) [54]	Retrospective cohort	n = 70 mixed sexes	NR					X	X					X		fair	The Swiss-Ski Power Test was not associated with a history of ACL injury
Senisik (2011) [55]	Prospective cohort	n = 109 males	control group: 23.8 (2.0)				X				X					fair	An increase in the tibial slope was associated with ACL injury
Sheehan (2012) [56]	Case–control	n = 40 mixed sexes	NR	X						X	X	X		X		fair	Landing with the centre of mass further posterior to the base of support was associated with ACL injury
Shimozaki (2018) [15]	Prospective cohort	n = 195 females	cases: 15.4 (0.3); controls: 15.5 (0.3)	X		X	X		X	X						fair	Increase in BMI and hip abductor muscle strength were associated with ACL injury
Smeets (2019) [12]	Prospective cohort	n = 46 females	cases: 21.02 (2.96); controls: 20.69 (3.19)					X			X	X	X			fair	ACL injury was positively associated with lateral hamstring activation during peak loading and the push off phase of a drop vertical jump
Smith (2012) [57]	Prospective cohort	n = 5047 mixed sexes	high school: 16.88 (1.17); college: 20.17 (1.34)					X		X	X		X	X		fair	ACL injury and LESS score were not associated
Steffen (2016) [58]	Prospective cohort	n = 880 females	20.9 (4.0)						X		X	X				fair	None of the five strength variables selected were associated with an increased risk of ACL injury
Steffen (2017) [59]	Prospective cohort	n = 838 females	21.0 (4.0)	X							X	X				fair	Balance was not associated with ACL injury
Webster (2019) [60]	Prospective cohort	n = 409 mixed sexes	17.2 (2)			X		X							X	fair	A flexion deficit or a side-to-side difference in anterior knee laxity was associated with ACL graft rupture
Westin (2018) [18]	Prospective cohort	n = 339 mixed sexes	cases: 17.6 (1.1); controls: 17.7 (1.2)			X	X	X						X		fair	ACL injury was positively associated with the left knee and athletes with fewer active years in skiing
Zazulak (2007) [61]	Prospective cohort	n = 277 mixed sexes	males: 19.3 (1.8); females: 19.4 (1.0)	X											X	fair	Lateral extension and flexion displacements of the trunk were associated with ACL injury
Zazulak (2007) [62]	Prospective cohort	n = 277 mixed sexes	males: 19.3 (1.8); females: 19.4 (1.0)	X											X	fair	Impaired core proprioception was associated with ACL injury in females but not in males
Zebis (2009) [17]	Prospective cohort	n = 55 females	24 (5)		X						X	X				fair	Reduced EMG preactivity of the semitendinosus and increased EMG preactivity of the vastus lateralis during side-cutting were associated with ACL injury

ACL, anterior cruciate ligament; BMI, body mass index; EMG, electromyography; FMS™, Functional Movement Screen; LESS score, Landing Error Scoring System; NR, not reported; PKAM, peak knee abduction moment; VDJ, vertical drop jump; 1RM, one-repetition maximum; ** male ACL injury with following graft rupture, 18.0 (2.3); male ACL injury with following no graft rupture, 18.5 (2.5); female ACL injury with following graft rupture, 15.9 (0.8); female ACL injury with following no graft rupture, 16.6 (1.2).

**Table 2:** Test categories for clinical use based current evidence.

Test Category	Recommended for Clinical Use?	Application Aspects	Current Limitations/Future Research Proposals
Joint Morphology and Anthropometrics	Yes	Attain an MRI scan to assess for femoral notch width (particularly in females at increased risk) [14,43,44] and, if applicable, tibia slope (in both sexes) [21,51]	Improved (e.g., real 3D) or alternative (less cost-intensive or more mobile) imaging modalities to assess geometric knee characteristics may help to better scale-up the application of such screening approaches in a wider range of athletes.
Balance and Postural Control	Yes, for specific purposes	Despite a clear conceptual link and preliminary evidence for a potential association with ACL (re)injury, there is considerable heterogeneity in the test procedures used and thus in the results available [19,42,50,56,61,62]. Due to a high standardizability, potentially helpful for progress tracking and tailored preventative interventions.	Exploring a more specific description of whole-body kinematics during dynamic movements as a complement to the assessment of general balance could be helpful in expanding our current understanding.
Jump Tests	Yes, for specific purposes	There is controversy whether there is an association between jump tests and ACL (re)injury [10,11,12,14,16,18,34,41,48,49] or not [13,30,35,39,40,54,57,60]. However, dynamic-knee-valgus- as well as landing-strategy-(i.e., joint angle and muscle activation) related screening tests have clear conceptual links to ACL (re)injury. Moreover, such tests may provide clinically relevant information to better tailor interventions and for progress tracking during training or return-to-sport.	Using 3D kinematics and kinetics for DVJ, consider more complex jumping tasks that require multiplane body movements including rotational components or single-leg landing strategies, as they occur during real-life sporting situations. On-field/in-game analyses may become more and more feasible based on the recent advances in measurement technology (e.g., wearable sensors coupled with deep-learning algorithms or combined with computer-vision approaches).
Strength Tests	Yes, for specific purposes	Despite a clear conceptual link and preliminary evidence for a potential association with ACL (re)injury, there is considerable heterogeneity in the test procedures used and thus in the results available [15,16,36,38,39,53]. Due to a high standardizability, potentially helpful for progress tracking and tailored preventative interventions.	Complementarily assessing the EMG activity during dynamic motion tasks and focusing on the intermuscular activation patterns rather than just using standard strength tests alone. It is not only a question of strength capacity but also timing and coordination of muscle activation.
Gait- and Running-related Tests	No	To date, there are only a few studies [17,31,39] and lacking evidence to support regular clinical use.	Use 3D instead of 2D assessment methods for research purposes, as movements relevant to ACL (re)injuries mainly occur in the anatomical sagittal plane and not in the global frontal plane.
Joint Laxity	No	Limited evidence for a potential association with the risk for ACL (re)injuries [14,15,33,46,60]. It is recommended using side-to-side comparisons of knee laxity over absolute measures if using such tests at all.	Examining data on active joint laxity while completing motion tasks such as vertical drop jumps (recent advances in measurement technology will pave the way, e.g., videofluoroscopy).

MRI, magnetic resonance imaging; ACL, anterior cruciate ligament; 2D, two-dimensional; 3D, three-dimensional; DVJ, drop vertical jump; EMG, electromyography.

## Data Availability

Data sharing is not applicable to this article.
